# Dispersion of Single-Walled Carbon Nanotubes by Aromatic Cyclic Schiff Bases via Non-Covalent Interactions

**DOI:** 10.3390/molecules29133179

**Published:** 2024-07-03

**Authors:** Lun Li, Pengfei Zhou, Jiali Wen, Panli Sun, Zongxia Guo

**Affiliations:** 1 Key Laboratory of Optic-Electric Sensing and Analytical Chemistry for Life Science, Ministry of Education, Shandong Key Laboratory of Biochemical Analysis, College of Chemistry and Molecular Engineering, Qingdao University of Science and Technology, Qingdao 266042, China; lilun0917@163.com (L.L.); mgfh587@163.com (P.Z.); wjl2211495487@126.com (J.W.); 2 College of Polymer Science and Engineering, Qingdao University of Science and Technology, Qingdao 266042, China; panlisun2017@163.com

**Keywords:** Schiff base, SWCNTs, π-π interaction, C-H·π interaction, lone pair·π interaction

## Abstract

One of the challenging issues that hinders the application of single-walled carbon nanotubes (SWCNTs) is the poor solubility and the inevitable formation of bundles. Efforts still need to be made towards solving the problem. Herein, we report a non-covalent strategy to disperse aggregated SWCNTs by aromatic cyclic Schiff bases assisted by ultrasonic techniques. The aromatic cyclic Schiff base (OMM) was synthesized via Schiff base reactions, and the molecular structure was determined by ATR-FT-IR, solid-state ^13^C-NMR, and HRMS. Although the yielded product showed poor solubility in aqueous solution and organic solvents, it could interact with and disperse the aggregated SWCNTs in dimethyl formamide (DMF) under the condition of ultrasound. UV-vis-NIR, FL, Raman spectra, AFM, and TEM, along with computer simulations, provide evidence for the interactions between OMM molecules and SWCNTs and the dispersion thereof. The semiconductive (7,5), (8,6), (12,1), and (9,7)-SWCNTs expressed a preference for dissolution. The capability of dispersion is contributed by π-π, C-H·π, and lone pair (lp)·π interactions between OMM and SWCNTs based on the simulated results. The present non-covalent strategy could provide inspiration for preparing organic cyclic compounds as dispersants for SWCNTs and then facilitate their further utilization.

## 1. Introduction

Single-walled carbon nanotubes (SWCNTs) have shown great potential applications in many materials with high performance, such as those found in the fields of optics, electronics, and photovoltaics [1,2,3,4,5,6]. One of the challenging issues that prevents the use of SWCNTs in a wide range of practical industrial applications is the poor solubility and inevitable formation of bundles [7,8]. Many efforts have been made to disperse SWCNTs to take advantage of their excellent natural properties. Non-covalent and covalent methods are often used, and excellent results have been achieved [9]. It is worth noting that non-covalent strategies could maintain the intrinsic nature of SWCNTs compared with covalent methods [10,11,12]. Surfactants [13], polymers [14], biomolecules [15], and π-conjugated small molecules [16] have been paid much attention, and electrostatic interactions [17], van der Waals interactions [18], π-π stacking [19], and donor–acceptor interactions [20] normally play a great role in exfoliating and debundling SWCNTs. In addition, an ultrasonic procedure was frequently applied to promote the dispersant–tube interactions and facilitate further exfoliation of bundled nanotubes [11,21]. 

As far as the non-covalent method, the π-conjugated small molecule is one class of promising compounds that could give rise to potential π-π interactions with SWCNTs [22]. Moreover, alternative functional groups could be decorated to offer additional electrostatic interactions, van der Waals interactions, etc., to further induce SWCNTs to disperse. Nogueira et al. reported that π-conjugated TPO molecules could realize the dispersion of SWCNTs through the synergistic effect of π-π stacking, charge transfer, and cationic-π interactions with an sp^2^ carbon skeleton [23]. Gao et al. showed that non-planar aromatic c-OBCB selectively interacted with certain chiral SWCNTs, and the adhesion of flexible side chains facilitated the interactions [24]. Chamorro et al. showed that cyclic tetramer nanorings, formed by aromatic π-conjugated molecules decorated with DNA bases via Watson–Crick base pairing rules, effectively unbundled SWCNTs, and π-π stacking and H-bonding played a great role [25]. In addition, PBI [26,27] and porphyrin [28,29] derivatives were also employed owing to their π-conjugated features, which can provide potential π-π interactions with SWCNTs. However, it is still imperative to solve the issue of how to prepare dispersed SWCNTs, although extensive scientific research has been persistently conducted. 

The aromatic cyclic Schiff base is a class of molecules potentially capable of the exfoliation of SWCNTs owing to their π-conjugated nature, although seldom research has been reported. Herein, we report an aromatic cyclic Schiff base (OMM) as a dispersant to explore the capability of exfoliating aggregated SWCNTs. OMM was synthesized via Schiff base reaction and contains four anthracene groups, which are linked by four C=N double bonds of the imines. It is expected that the π-conjugated structures of these molecules could offer the ability of affinity to the surface of bundles of SWCNTs and further induce the exfoliation of the nanotubes. UV-vis-NIR absorption spectroscopy, Fluorescence spectroscopy, and Raman spectroscopy were adopted to characterize the optical properties. The atomic force microscope (AFM) and transmission electron microscopy (TEM) were performed to detect the nanostructures of SWCNT dispersions. Computer simulation results proposed evidence of why and how OMM molecules could interact with SWCNTs.

## 2. Results

### 2.1. Synthesis of OMM

Firstly, the dispersant OMM was synthesized according to the reported method [30], and the molecular structure was confirmed. Then, the capability of OMM to disperse bundled SWCNTs was explored. The interactions between OMM molecules and SWCNTs were simulated to give insight into the mechanism, mainly based on the view of energy. 

**Scheme 1:**
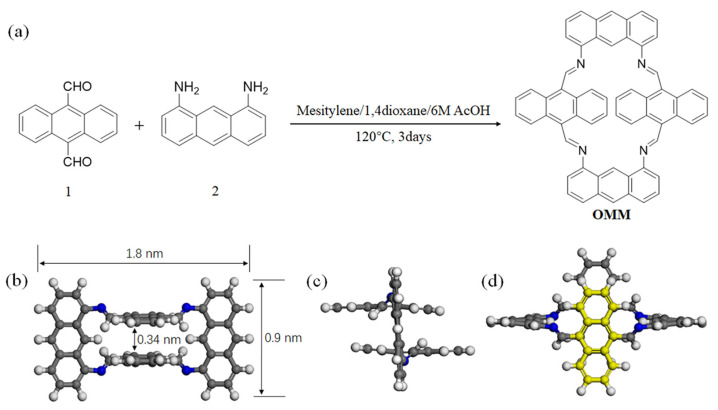
(**a**) Synthesis of OMM molecule; (**b**–**d**) views of optimized OMM structures from different directions. Atom types are marked by different colors (gray: carbon; blue: nitrogen; white: hydrogen). The yellow atoms belonging to the upper anthracene group are marked to indicate the different plane directions compared to the lower anthracene group.

The OMM was synthesized according to reported procedures by Schiff base reaction of 9,10-anthracenedicarbaldehyde and 1,8-diaminoanthracene (Scheme 1a). The obtained rufous product showed poor solubility in organic solvents and water and is slightly soluble in dimethyl formamide (DMF), dichloromethane (DCM), tetrahydrofuran (THF), dimethyl sulfoxide (DMSO), and trifluoroisopropanol. The poor solubility is likely due to the strong π-π interactions among anthracene groups. Due to the poor solubility of OMMs, the ^1^H NMR and ^13^C NMR in solution were tried, but authentic and dependable data were not acquired.

**Figure 1:**
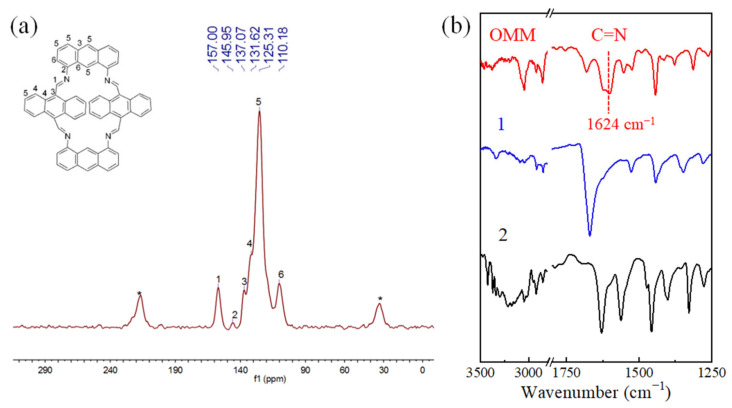
(**a**) Solidstate ^13^C CP/MAS NMR spectrum of OMM; (**b**) ATRFTIR spectra of 9,10anthracenedicarbaldehyde (1), 1,8diaminoanthracene (2), and OMM.

Attenuated total reflection Fourier transform infrared spectroscopy (ATR-FT-IR), solid-state ^13^C CP/MAS NMR spectra, and high-resolution mass spectrometry (HRMS) were carried out to confirm the molecular structure. In FT-IR spectra, the disappearance of the band at 3400–3200 cm^−1^ was due to the N–H stretching vibration, while the presence of the strong peak at 1624 cm^−1^ was ascribed to the C=N stretching vibration [31], which provided convincing evidence for the formation of imine linkages. For OMM (Figure 1a), the peak at around 3040 cm^−1^ corresponds to the aromatic =C–H stretching vibration [32]. In solid-state ^13^C CP/MAS NMR spectra, the major peaks observed at 145.95, 137.07, 131.62, 125.31, and 110.18 ppm could be attributed to the aromatic carbon atoms (* means spinning sidebands in this article) and the peak assignment can be found in the inset in Figure 1a. The peak at 157.00 ppm could be attributed to the carbon atoms present in the imine bond. Based on the analysis of the FT-IR and NMR results, the Schiff-base reaction was successful. For HRMS, OMM calcd. for C_60_H_37_N_4_ [M+H]^+^: 813.3013; Found: 813.3016 (Figure S1). An aromatic cyclic Schiff base was obtained, as shown in Scheme 1, based on the consistency between the calculated and theoretical value of molecular weight. From the optimized structures, as shown in Scheme 1b–d, the length of OMM is about 1.8 nm, and the length of the anthracene group is about 0.9 nm (Scheme 1b). The two anthracene groups originating from 9,10-anthracenedicarbaldehydes are parallel but not overlapped and clearly prefer to be close to each other with a distance of 0.34 nm, which is the most appropriate distance for π-π interactions (Scheme 1b,c). It is clear that the anthracene groups from 1,8-diaminoanthracene are parallel and in the same plane (Scheme 1c,d).

### 2.2. Characterizations of SWCNT Dispersions

The capability of the exfoliation of SWCNTs by OMM was then explored. DMF was selected as the solvent because of the low solubility of OMM. The weight ratio of SWCNTs to OMM was tested, ranging from 1:1 to 1:20. From the UV-vis-NIR spectra (Figure S2), it was found that the weight ratio of 1:15 of SWCNTs to OMM is reasonable for dispersing SWCNTs, and it is obvious that the absorption band from 900 to 1400 nm belonging to semiconductive SWCNTs turned out to be sharper and more resolved. In addition, two other organic solvents, methylbenzene and isopropanol, were used, and DMF was proved to be the best solvent for dispersion (Figure S3). The concentration of SWCNTs was 0.05 mg/mL, and the weight ratio of SWCNTs to OMM was 1:15 for all the following experiments.

**Figure 2:**
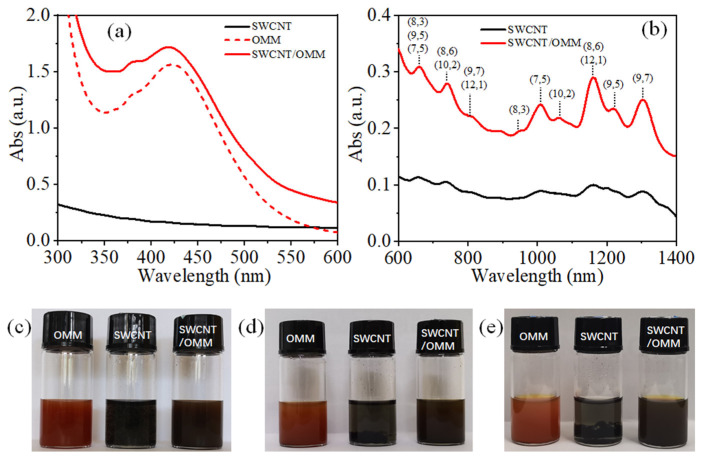
Absorption spectra for SWCNT, OMM, and SWCNT/OMM dispersions in DMF: (**a**) the obvious absorption bands from 300 to 600 nm belong to OMM molecules; (**b**) the obvious absorption bands from 600 to 1400 nm are from dispersed SWCNTs; (**c**) photo of samples just after ultrasonication; (**d**) photo of samples 8 h after ultrasonication; and (**e**) photo of samples 75 h after ultrasonication.

In Figure 2a, the OMM showed absorption centered at 421 nm, which could be attributed to the π-π* transition of the conjugated aromatic structure [33]. No obvious absorption was observed for the raw SWCNTs. For the dispersion of SWCNT/OMM, the band at 421 nm blue-shifted to 418 nm, indicating the formation of π-π stacking interactions between OMM and SWCNT [34]. In Figure 2b, characteristic peaks for raw SWCNTs were found at 657/1010, 737/1161, and 809/1304 nm, which can be assigned to (7,5)-SWCNT, (8,6), (12,1)-SWCNT, and (9,7)-SWCNT [35]. For SWCNT/OMM, the band peaks belonging to the S_11_ transition of SWCNT slightly shifted to 1008, 1160, and 1303 nm with normalized bandwidths of 1181, 582, and 765 nm/Abs., respectively [36]. Compared to the large values of 6307, 3628, and 2987 nm/Abs. for raw SWCNTs, the number of single SWCNTs was enhanced in SWCNT/OMM dispersions, suggesting that OMM molecules could interact with bundled nanotubes and disperse them into single ones [37]. So, the capability of OMM molecules to exfoliate aggregated SWCNTs was basically confirmed. 

**Figure 3:**
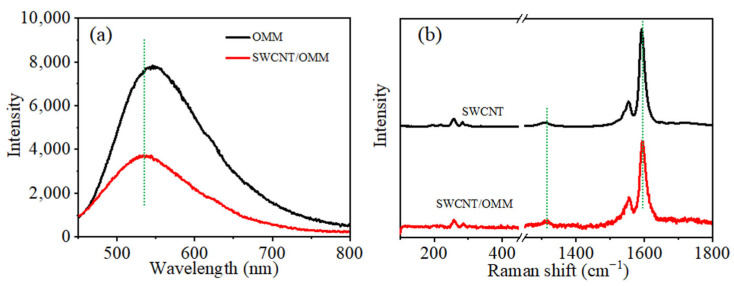
(**a**) Fluorescence spectra in DMF with λ_ex_ = 421 nm; (**b**) Raman spectra recorded at 633 nm excitation wavelength.

From the FL spectra in Figure 3a, the emission band found at 547 nm was attributed to OMM molecules. Such OMM emission shifted to 536 nm, and there was a blue band shift of 11 nm in SWCNT/OMM dispersions. In addition, the emission intensity of the OMM was reduced greatly, and the quenching efficiency was calculated as 52% for SWCNT/OMM. The band shift, along with the emission quenching, declared the conceivable existence of interactions between OMM and SWCNTs [38,39]. Raman spectra, recorded at 633 nm excitation wavelength for SWCNT and SWCNT/OMM, were conducted to further validate the affinity of OMM molecules to SWCNT. The D band peaks were recorded at 1308 and 1312 cm^−1^, and the G band peaks were found at 1592 and 1594 cm^−1^ for raw SWCNT and SWCNT/OMM, respectively. The red shift of the G band may be attributed to the n-type charge transfer from the OMM to SWCNTs [40]. The I_D_/I_G_ value for SWCNT/OMM was 0.077, which exhibited no distinct variation with respect to the 0.072 value from raw SWCNTs. It was deduced that the treatment of raw SWCNTs with the addition of OMM molecules through ultrasonic techniques in the present work would not generate defects for SWCNTs [41,42], and the affinity of OMMs to SWCNTs originated from non-covalent interactions instead of covalent bonds.

**Figure 4:**
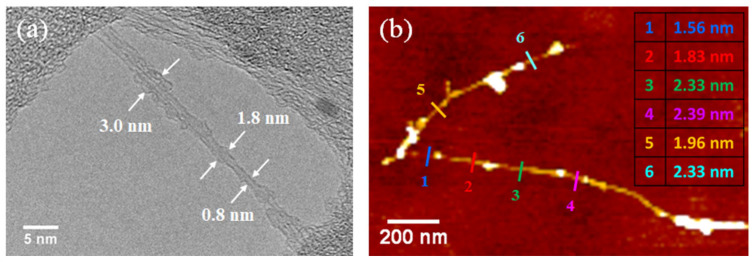
Images recorded from SWCNT/OMM dispersions: (**a**) TEM; (**b**) AFM; inset shows the heights of nanotubes with lines marked.

TEM and AFM images were recorded for the SWCNT/OMM dispersions. For the commercial HiPco SWCNTs with a broad chirality range, the diameter of a single nanotube ranges from about 0.8 to 1.2 nm. In Figure 4a, a single single-walled carbon nanotube was observed based on the indicated diameter of 0.8 nm. The protuberances adsorbed on the side wall of the nanotube were clearly seen, and they could increase the diameter of the nanotube up to 3.0 nm. Considering that the addition of OMM could improve the dispersibility of SWCNTs and the dimension of OMM, the protuberances might be the adsorbed ACBS molecules or aggregates, although it could not be excluded that some protrusions might be amorphous carbon. The AFM image in Figure 4b showed single nanotubes, and the values from about 1.6 to 2.4 nm in the inset are the heights of the positions with lines marked. The height from AFM is basically consistent with the width from TEM. Compared to the TEM images of the pristine SWCNTs with diameters larger than 10 nm (Figure S4), it was speculated that the OMM molecules preferred to interact with SWCNTs and adsorbed on the side wall, resulting in the generation of separated single-walled carbon nanotubes [42].

## 3. Discussion

**Figure 5:**
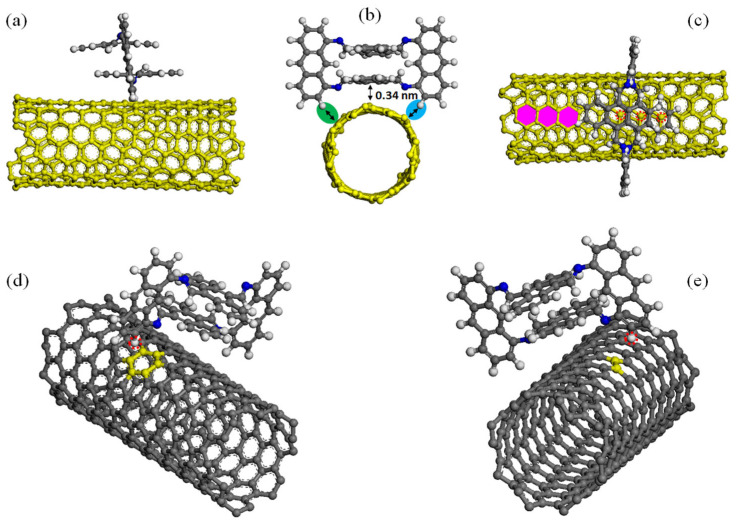
(**a**–**e**) Views of a geometry-optimized complex formed by an OMM and an (8,6)-SWCNT from different orientations. (**b**) The distance between SWCNT and the anthracene group of OMM close to SWCNT; (**c**) three purple regular hexagons overlapped on hexatomic rings of hybridized sp^2^ carbons are approximately indicating the long-axis direction of SWCNT. Three carbons of nanotube marked by red dotted circles show the relative locations with respect to the anthracene group close to SWCNT; (**d**,**e**) the hydrogen atom closest to SWCNT is marked by a red dotted circle to clearly show the relative locations of (**d**) the yellow π-conjugated hexatomic rings of hybridized sp^2^ carbons or (**e**) the yellow C=C bond of SWCNT. (**d**) and (**e**) show the green and blue elliptic region in (**b**), respectively.

To understand the dispersion ability of OMM molecules and the non-covalent interactions between OMMs and SWCNTs, geometry optimization simulations were performed [43]. An (8,6)-SWCNT was used for simulation, considering the results from experiments. Different relative arrangements of the OMM and (8,6)-SWCNT were simulated, and the system showing the lowest energy will be discussed. Figure 5 shows some views of a simulated complex formed by an OMM and a (8,6)-SWCNT from different orientations. The distance between the SWCNT and the anthracene group of the OMM parallel and close to the SWCNT is 0.34 nm, which is the most appropriate for π-π stacking interactions. The long-axis orientation of this anthracene group is basically along the long-axis direction of the SWCNT (Figure 5c). In addition, the sp^2^ carbons of the SWCNT are almost right under the centers of the benzene rings of the anthracene group. Such a relative arrangement of OMMs and SWCNTs could increase the π-π interaction. In Figure 5d, the distances between the hydrogen atom from the C-H bond and the six hybridized sp^2^ carbons of the π-conjugated hexatomic rings of the SWCNT were measured, and the six values are each nearly 0.30 nm. It is clearly suggested that this hydrogen atom is just above the center of the π-conjugated hexatomic rings of the SWCNT. For the other hydrogen atom close to the SWCNT (Figure 5e), it was observed that the hydrogen atom is right above the center of a C=C bond since the distances between the hydrogen atom and the two carbons are both 0.27 nm. Carbon atoms around this C=C bond are all far away from this hydrogen (>0.3 nm). So, besides the π-π stacking, C-H·π interactions were proposed to contribute to the formation of complexes as well. Due to the band shifts from UV-vis-NIR, FL, and Raman spectra, it is deduced that electron transfer from electron-rich OMMs to electron-deficient SWCNTs could occur [38,40,42]. As shown in Figure 5b, two nitrogen atoms from C=N imine bonds are close to the side wall of the SWCNT with a distance of about 0.33 nm, suggesting the possible existence of lone pair (lp)·π interactions between nitrogen and π-conjugated carbon nanotubes [44,45]. The energy of one complex was calculated as 13,133.68 kcal/mol, showing an energy decrease of 32.27 kcal/mol compared to the sum of an isolated SWCNT (12,373.11 kcal/mol) and OMM (792.84 kcal/mol). It can be deduced that the reduced value of system energy would be larger if one SWCNT adsorbed more OMM molecules, as indicated in Figure 4a. Based on the discussions above, OMM molecules could adsorb on the side wall of the nanotubes mainly via non-covalent interaction, that is, π-π stacking, C-H·π interactions, and lp·π interactions, and the formation of complexes could be driven by the deduced energy of the system, resulting in the dispersion of bundled SWCNTs into single ones. The possibilities of forming complexes, including one SWCNT and two OMMs or one OMM with two SWCNTs, were simulated and confirmed (Figures S5 and S6), indicating the diversity of the complexes in dispersions.

Desorption experiments have been tried by rinsing the SWCNT/OMM with DMF, however, the desorption of OMMs from SWCNTs could not be confirmed from the XPS spectra (Figure S7). This could be due to the poor solubility of OMMs and SWCNTs, which can both aggregate and then precipitate as a mixture even after the desorption of OMMs from SWCNTs.

The present findings could bring some inspiration for designing organic molecules for dispersing SWCNTs, especially aromatic cyclic molecules. Research on exploiting SWCNTs by a series of aromatic cyclic Schiff bases is ongoing to understand the corresponding mechanism and for the purpose of applications of dispersed SWCNTs.

## 4. Materials and Methods

### 4.1. Materials

HiPco SWCNTs with a broad chirality range (0.8−1.2 nm in diameter, 100−1000 nm in length, >65% of carbon as SWCNTs, Batch No. HR37–075A) were purchased from XFNANO (Nanjing, China). DMF (AR, >99.5%) was purchased from Sinopharm Chemical Reagent Co., Ltd. (Suzhou, China). Other reagents and solvents were purchased from commercial sources and used without further purification unless otherwise stated.

The synthesis of OMM was carried out according to a previous report [30]. 1,5-Diaminoanthracene was synthesized according to the literature [46]. A mixture of 1,8-diaminoanthracene (62.4 mg, 0.3 mmol) and 9,10-Anthracenedicarbaldehyde (70.2 mg, 0.3 mmol) was mixed with mesitylene/1,4-dioxane/6M AcOH (19/1/2 by volume; 16.5 mL) in a heavy-wall pressure reactor. Next, the reactor was degassed by three freeze-pump-thaw cycles and purged with nitrogen. The reactor was sealed and heated at 120 °C for 3 days under undisturbed conditions. When the system was cooled to room temperature, the product was collected by centrifugation, washed with anhydrous THF and acetone, and then dried under dynamic vacuum at 120 °C for 10 h to yield a rufous powder (61 mg, 75%).

### 4.2. General Procedure for Dispersion Preparations of SWCNTs and OMM-SWCNTs

*SWCNTs*: 0.25 mg of SWCNTs were added to 5 mL of DMF, followed by bath ultrasonic treatment (100 W, 45 KHz, 25 °C) for 1 h, and then the samples were treated by centrifugation (10,000 rpm) for 1 h; 80% supernatant was taken for further ultrasonic treatment for 1 h and centrifugated for 30 min, and 80% supernatant was used for the following characterizations.

*OMM-SWCNTs*: 0.25 mg of SWCNTs and 3.75 mg OMM (weight ratio, 1:15) were mixed into 5 mL of DMF. The treatment procedures of samples are the same as that of *SWCNTs*.

### 4.3. Characterizations

OMM. Fourier transform infrared spectroscopy was carried out with a BRUKERHQL005 FT-IR spectrometer (Bruker, Germany) and Bruker Tensor 27 (Bruker, Germany) in the 400–4000 cm^−1^ region by using the attenuated total reflection (ATR). Solid-state ^13^C NMR cross-polarization (CP) spectra were performed on an Agilent 600 DD2 spectrometer (Agilent, Santa Clara, CA, USA) at a resonance frequency of 150.45 MHz. ^13^C CP/MAS NMR spectra were recorded with a spinning rate of 15 KHz, with a 4 mm probe at room temperature, with a delay time of 3 s and a contact time of 2 ms. Mass spectral data were obtained by using a DCTB matrix-assisted laser desorption/ionization time-of-flight mass spectrometry (MALDI-TOF MS, Bruker, Germany) instrument.

Dispersions. A total of 30 min of ultrasonic treatments for the objective supernatants in Section 4.2 were performed before spectral characterizations and sample preparation for morphological observation. Suspensions for TEM and AFM measurements were diluted 5 times with DMF before drop-casting onto the substrates. The UV-vis-NIR absorption spectra were measured using a Cary 5000 spectrometer (Agilent) and 10 × 10 mm quartz cuvettes. The FL spectra were obtained with an RF-6000 Fluorescence spectrometer (Shimadzu, Japan) using a 150 W Xenon lamp for detection. For Raman and AFM characterization, the samples were drop-casted onto silica wafers and dried at 70 °C overnight under atmospheric pressure in an oven. The Raman spectra were acquired with an inVia InSpect confocal Raman microscope (Renishaw, Wotton-under-Edge, UK), and a laser with an output of 633 nm was used for sample excitation. Silicon wafers with samples were settled on metal disks with double-faced adhesive tape, and AFM measurements were carried out with a MULTIMODE8 scanning probe microscope (Bruker, Germany). Suspensions were drop-casted onto Microgrid carbon grids, and TEM was conducted on a JEM-2100PLUS (Nippon Electronics Corporation, Tokyo, Japan) with an acceleration voltage of 200 kV.

### 4.4. Computational Simulation

The geometry optimizations and calculations of energies were performed by using the COMPASSII force field [43]. The cutoff distance was set to 12.5 Å, the spline width was set to 1 Å, and the buffer width was set to 0.5 Å. The convergence criteria were as follows: maximum iterations, 5000; energy, 0.001 kcal/mol; force, 0.5 kcal/mol/Å. The period and the solvent DMF were not taken into account in the simulation. 

The formation energies *ΔE*_OMM+SWCNT_ for the non-covalent complex of OMMs with SWCNTs were calculated according to the general equation:

*ΔE*_OMM+SWCNT_ = *E*_OMM+SWCNT_ − (*E*_OMM_ + *E*_SWCNT_)

where *Ei* is the absolute energy of the corresponding compound.

## 5. Conclusions

In summary, an aromatic cyclic Schiff base (OMM) was designed, and its capability of dispersing aggregated SWCNTs has been systematically investigated using UV-vis-NIR, FL, Raman spectra, AFM, and TEM, along with computer simulation methods. It was found that OMMs could interact with SWCNTs via π-π, C-H·π and lone pair (lp)·π interactions, which are generated between the anthracene group, the C-H bonds, and the N atoms from an OMM close to the side wall of an SWCNT and the π-conjugated carbon nanotubes. The OMM/SWCNT complexes show decreased system energy with respect to the sum energy of corresponding isolated ones. The intermolecular non-covalent interactions and the energy variation might be the driving forces for exploiting the bundled SWCNTs into separated ones. The present non-covalent strategy could give some insights into the design of dispersants, especially the organic cyclic Schiff base derivatives, for preparing dispersed SWCNTs. 

## Data Availability

The data in this study are included in this article and Supplementary Materials. Further inquiries can be directed to the corresponding authors.

## Supplementary Materials

The following supporting information can be downloaded at https://www.mdpi.com/article/10.3390/molecules29133179/s1: Figure S1: HRMS spectrum of OMM; Figure S2: UV-vis-NIR spectra of SWCNT/OMM dispersions with different weight ratios in DMF; Figure S3: UV-vis-NIR spectra of SWCNT/OMM dispersions in different organic solvents with a weight ratio 1:15 of SWCNT to OMM; Figure S4: TEM images of pristine SWCNTs; Figure S5: A geometry optimized complex including one SWCNT and two OMMs; Figure S6: A geometry optimized complex including two SWCNTs and one OMM; Figure S7: N1s narrow-scan XPS spectra of SWCNT/OMM and SWCNT/OMM after rinsing with DMF.
