# Supplementary material for: Dispersion of Single-Walled Carbon Nanotubes by Aromatic Cyclic Schiff Bases via Non-Covalent Interactions
Source: Molecules. 2024 Jul 3;29(13):3179. doi: 10.3390/molecules29133179 (PMC11243016; doi:10.3390/molecules29133179)
Supplement: Supplementary file 1 [file molecules-29-03179-s001.zip › molecules-3048474-supplementary.pdf]

Supplementary Materials

Dispersion of Single-Walled Carbon Nanotubes by Aromatic Cyclic Schiff Bases via Non-Covalent Interactions

Lun Li [1], Pengfei Zhou [1], Jiali Wen [1], Panli Sun [2] and Zongxia Guo [1,*]

[1] Key Laboratory of Optic-Electric Sensing and Analytical Chemistry for Life Science, Ministry of Education, Shandong Key Laboratory of Biochemical Analysis, College of Chemistry and Molecular Engineering, Qingdao University of Science and Technology, Qingdao 266042, China; lilun0917@163.com (L.L.); mgfh587@163.com (P.Z.); wjl2211495487@126.com (J.W.)

[2] College of Polymer Science and Engineering, Qingdao University of Science and Technology, Qingdao 266042, China; panlisun2017@163.com

* Correspondence: zxguo@qust.edu.cn (Z.G.)

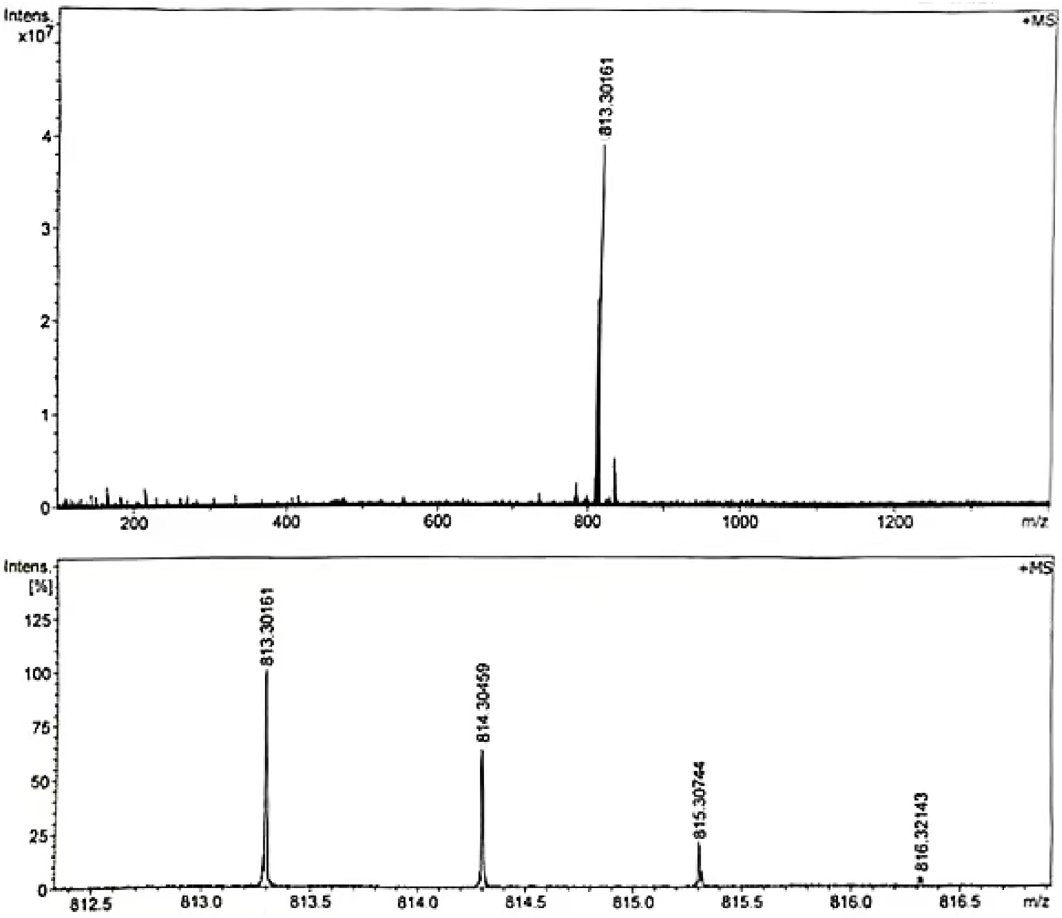

Figure S1. HRMS spectrum of OMM.

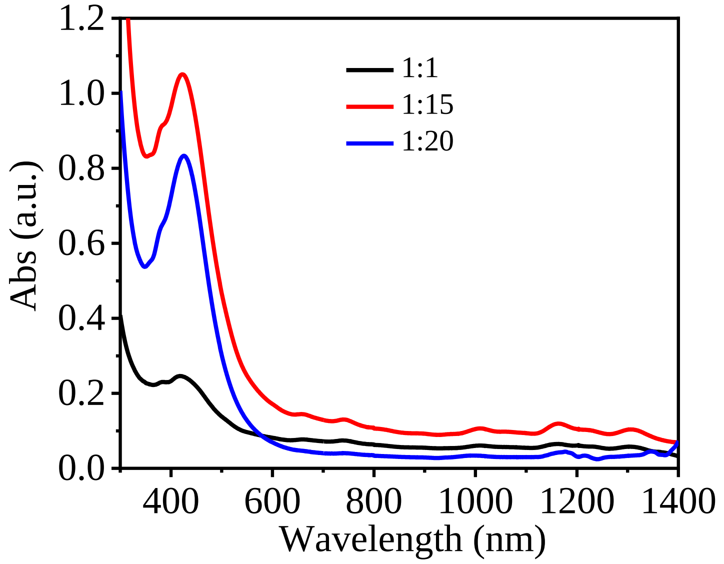

Figure S2. UV-vis-NIR spectra of SWCNT/OMM dispersions with different weight ratios in DMF.

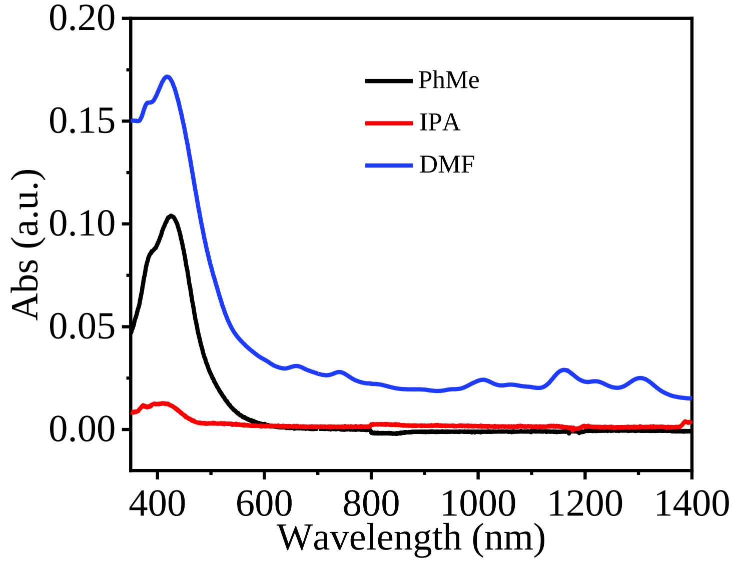

Figure S3. UV-vis-NIR spectra of SWCNT/OMM dispersions in different organic solvents with a weight ratio 1:15 of SWCNT to OMM.

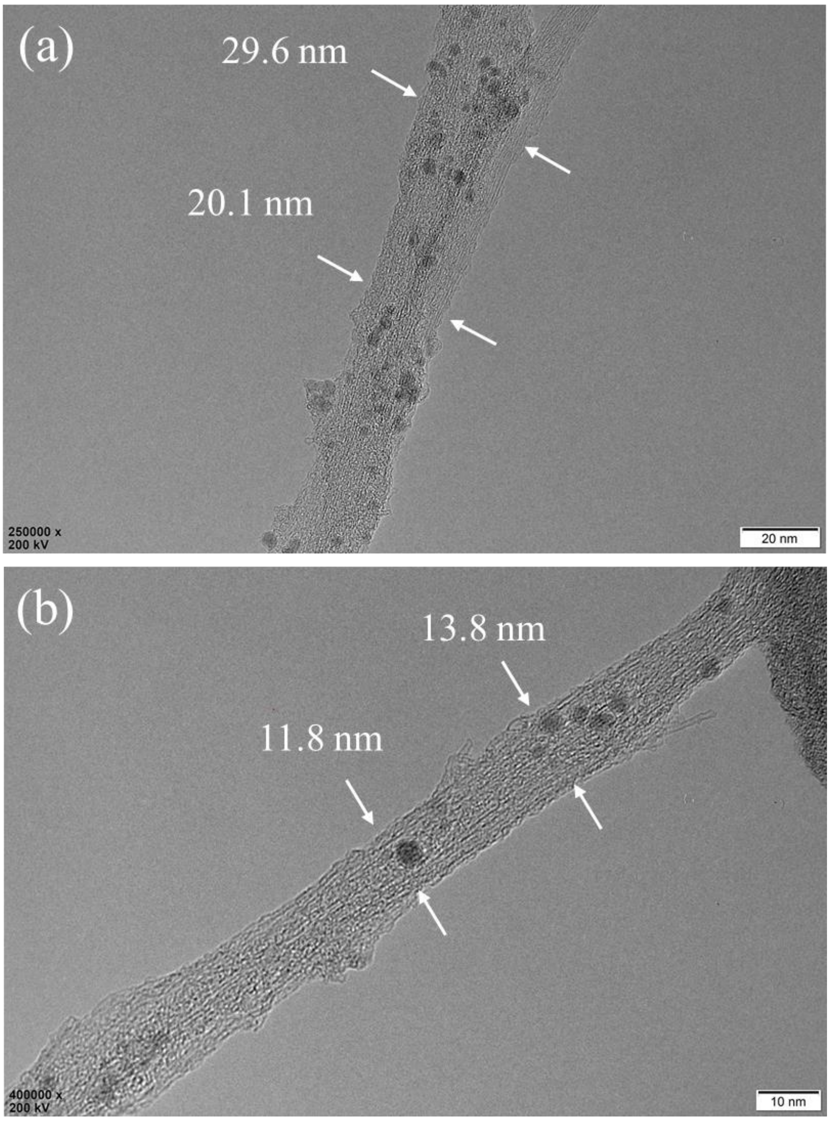

Figure S4. TEM images of pristine SWCNTs.

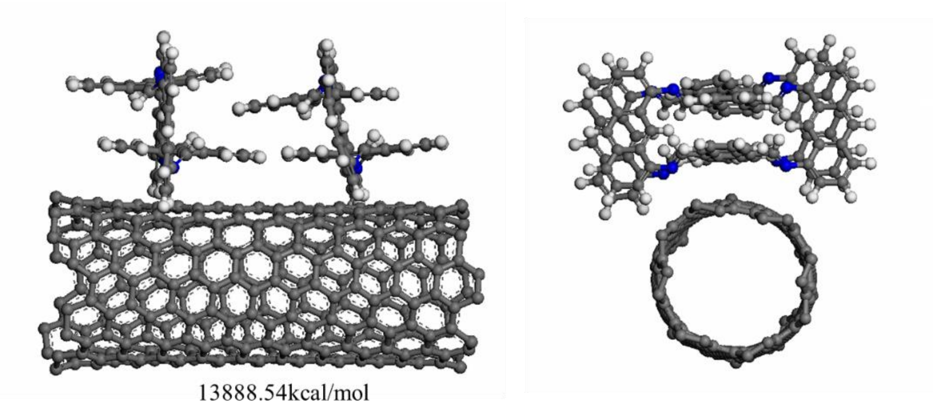

$$\triangle E_{SWCNT/OMM/OMM} = E_{SWCNT/OMM/OMM} - E_{SWCNT} - 2E_{OMM} = -70.25 \text{ kcal/mol}$$

Figure S5. A geometry optimized complex including one SWCNT and two OMMs.

Figure S5 shows one possible configuration of a complex including one SWCNT and two OMMs. π-π stacking between two OMMs could be observed. The decrease of the complex energy compared to that of all monomers indicates the tendency of complex formation.

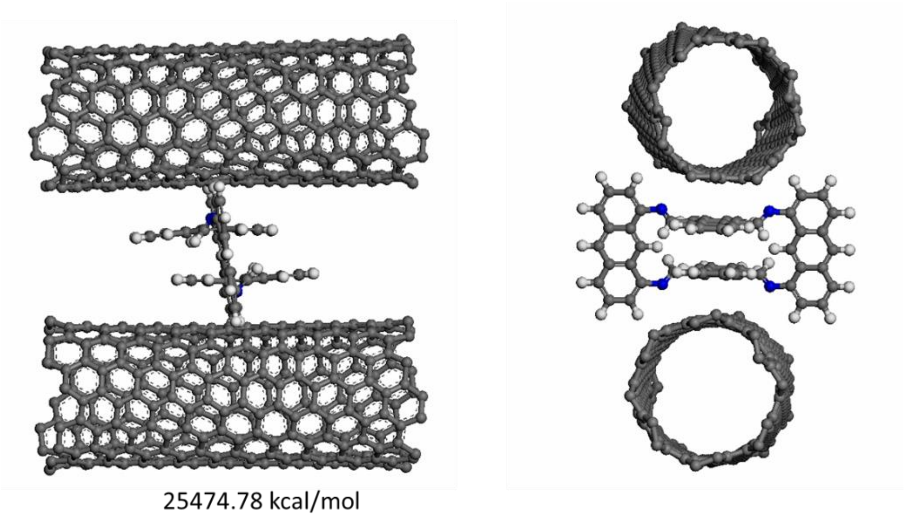

$$\triangle E_{SWCNT/OMM/SWCNT} = E_{SWCNT/OMM/SWCNT} - 2\times E_{SWCNT} - E_{OMM} = - 64.28 \text{ kcal/mol}$$

Figure S6. A geometry optimized complex including two SWCNTs and one OMM.

Figure S6 shows one possible configuration of a complex including two SWCNTs and one OMM. The interactions between the OMM and any one SWCNT are the same. It could be suggested that it was due to the symmetrical molecular structure. The decrease of the complex energy compared to that of all monomers indicates the tendency of complex formation.

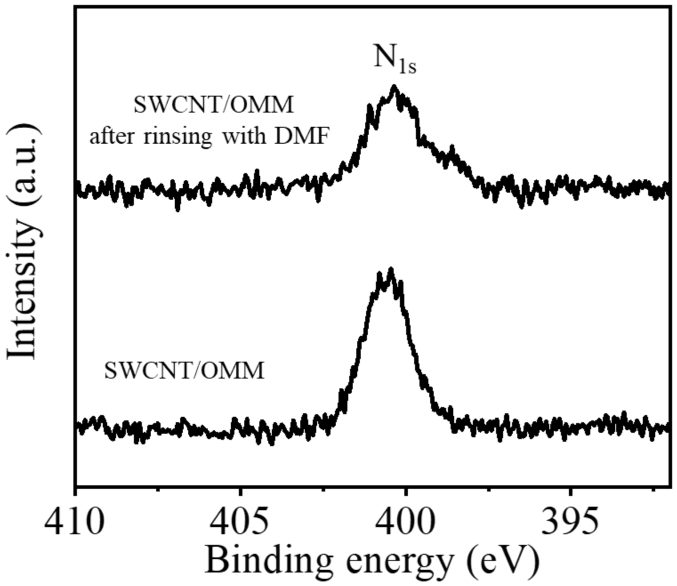

Figure S7. N_{1s} narrow-scan XPS spectra of SWCNT/OMM and SWCNT/OMM after rinsing with DMF.
